# Three rounds (1R/2R/3R) of genome duplications and the evolution of the glycolytic pathway in vertebrates

**DOI:** 10.1186/1741-7007-4-16

**Published:** 2006-06-06

**Authors:** Dirk Steinke, Simone Hoegg, Henner Brinkmann, Axel Meyer

**Affiliations:** 1 Lehrstuhl für Evolutionsbiologie und Zoologie, Department of Biology, University of Konstanz, 78457 Konstanz, Germany; 2 Département de biochimie. Université de Montreal, Montreal, QC, H3C3J7, Canada; 3 Canadian Centre for DNA Barcoding, Biodiversity Institute of Ontario, University of Guelph, Guelph, ON, N1G 2W1, Canada

## Abstract

**Background:**

Evolution of the deuterostome lineage was accompanied by an increase in systematic complexity especially with regard to highly specialized tissues and organs. Based on the observation of an increased number of paralogous genes in vertebrates compared with invertebrates, two entire genome duplications (2R) were proposed during the early evolution of vertebrates. Most glycolytic enzymes occur as several copies in vertebrate genomes, which are specifically expressed in certain tissues. Therefore, the glycolytic pathway is particularly suitable for testing theories of the involvement of gene/genome duplications in enzyme evolution.

**Results:**

We assembled datasets from genomic databases of at least nine vertebrate species and at least three outgroups (one deuterostome and two protostomes), and used maximum likelihood and Bayesian methods to construct phylogenies of the 10 enzymes of the glycolytic pathway. Through this approach, we intended to gain insights into the vertebrate specific evolution of enzymes of the glycolytic pathway. Many of the obtained gene trees generally reflect the history of two rounds of duplication during vertebrate evolution, and were in agreement with the hypothesis of an additional duplication event within the lineage of teleost fish. The retention of paralogs differed greatly between genes, and no direct link to the multimeric structure of the active enzyme was found.

**Conclusion:**

The glycolytic pathway has subsequently evolved by gene duplication and divergence of each constituent enzyme with taxon-specific individual gene losses or lineage-specific duplications. The tissue-specific expression might have led to an increased retention for some genes since paralogs can subdivide the ancestral expression domain or find new functions, which are not necessarily related to the original function.

## Background

**Figure 1:**
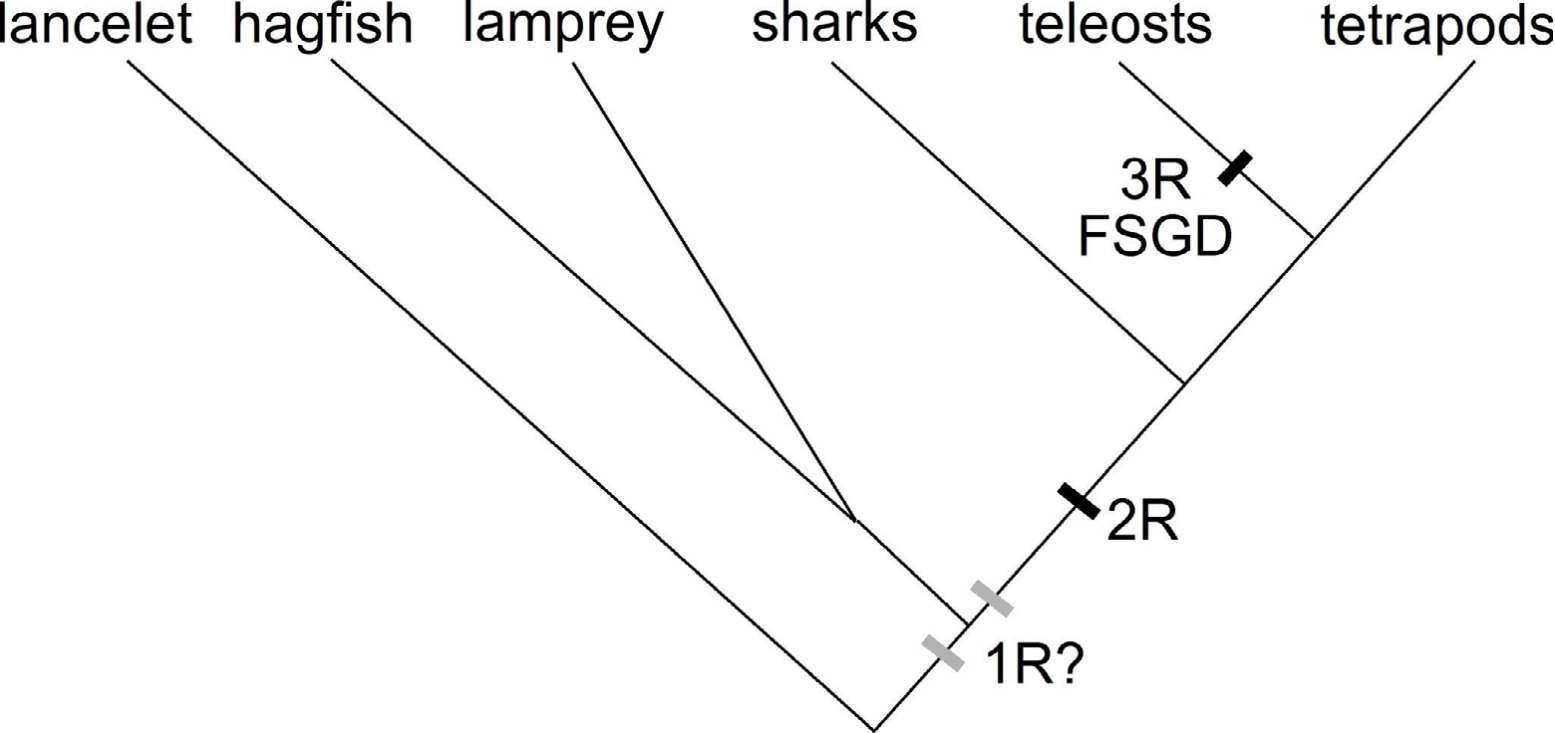
General overview of phylogenetic relationships among gnathostomes and the proposed phylogenetic timing of genome duplication events. Grey rectangles depict the possible position of the first genome duplication (1R); the black ones show the second genome duplication (2R), and fish-specific genome duplication (FSGD or 3R).

In many cases, evolution is accompanied by an increase of genetic and phenotypic complexity, yet the biochemical machinery necessary for the energy supply of an increasing diversity of cell- and tissue types had to work effectively, even if different tissues have specific conditions such as pH values, ion and substrate concentrations. Based on basic data such as genome sizes and allozymes, Ohno [1] proposed that the increase in complexity-during the evolution of the vertebrate lineage was accompanied by an increase in gene number due to duplication of genes and/or genomes. Recent data from genome sequencing projects showed that genome size is not strongly correlated with the numbers of genes an organism possesses. Nevertheless, for many genes, multiple copies can be found in vertebrates, while basal deuterostomes and invertebrates typically have only one orthologous copy. The "one-two-four" rule is the current model to explain the evolution of gene families and of vertebrate genomes more generally (Figure 1). Based on this model, two rounds of genome duplication occurred early in the vertebrate evolution [2,3], but see also [4,5]. An ancestral genome was duplicated to two copies after the first genome duplication (1R), and then to four copies after the second (2R) duplication [6,7]. While it is commonly accepted that 1/2R occurred before the divergence of Chondrichthyes [8], the position of lamprey and hagfish relative to the 1R still remains unclear, even though there is some evidence for a 1R-early (before divergence of cyclostomes) [9]. Recent data suggest that an additional whole genome duplication occurred in the fish lineage (3R or fish-specific genome duplication, extending the "one-two-four" to a "one-two-four-eight" rule [10-16].

Duplicated genes, resulting from large scale duplications, initially possess the same regulatory elements and identical amino-acid sequence and are therefore thought to be redundant in their function, which means that inactivation of one of the two duplicates should have little or no effect on the phenotype, provided that there are no dosage compensation effects [17]. Therefore, since one of the copies is free from functional constraint, mutations in this gene might be selectively neutral and will eventually turn the gene into a non-functional pseudogene. Although gene loss is a frequent event, 20–50% of paralogous genes are retained for longer evolutionary time spans after a genome duplication event [18,19]. On the other hand, a series of non-deleterious mutations might change the function of the duplicate gene copy [20]. Natural selection can prevent the loss of redundant genes [21] if those genes code for components of multidomain proteins, because mutant alleles disrupt such proteins. A selective advantage due to a novel function might be sufficient to retain this gene copy and to select against replacement substitutions and prevent this functional gene copy from turning into a pseudogene. In this way, genes can pick up new functions (neofunctionalization) [6] or divide the ancestral function between the paralogs (subfunctionalization) [22].

The glycolytic pathway is particularly suitable for testing theories of enzyme evolution and the involvement of gene/genome duplications. Previous phylogenetic analyses of these enzymes mainly focused on deep phylogenies [23,24] or the evolution of alternative pathways in different organisms, which displays high variability in bacteria [25,26]. This central metabolic pathway is highly conserved and ancient; it is therefore possible to compare enzymes from phylogenetically distant organisms [27]. The standard pathway includes 10 reaction steps; glucose is processed to pyruvate with the net yield of two molecules of adenosine triphosphate and two reduced molecules of hydrogenated nicotinamide adenine dinucleotide per molecule of glucose broken down. The classical glycolytic reactions are catalyzed by the following 10 enzymes: hexokinase (HK; EC 2.7.1.1), phosphoglucose isomerase (PGI; EC 5.3.1.9), phosphofructokinase (PFK; EC 2.7.1.11), fructose-bisphosphate aldolase (FBA; EC 4.1.2.13); triosephosphate isomerase (TPI; EC 5.3.1.1), glyceraldehyde-3-phosphate dehydrogenase (GAPDH; EC 1.2.1.12), phosphoglycerate kinase (PGK; EC 2.7.2.3), phosphoglycerate mutase (PGAM; EC 5.4.2.1), enolase (ENO; EC 4.2.1.11), and pyruvate kinase (PK; EC 2.7.1.40) [28].

The tertiary structures of all 10 of these enzymes show a superficial similarity; they are all variations on a common theme [27]. All glycolytic enzymes belong to the class of α/β-barrel proteins. Since this pathway is of crucial importance for the energy delivery of any cell, these genes are thought to be highly conserved and therefore have often been used as phylogenetic markers for "deep" phylogenies [23,29,30]. In fact, glycolytic enzymes are probably among the most conserved proteins known. Many vertebrate genes occur in multiple copies in the genome, and are often expressed in a tissue-specific manner. This increased genetic complexity might be utilized for highly specific requirements in terms of substrate optimum, pH value and salt concentration in different types of tissues [31]. Glucokinase, one of the hexokinase isozymes, is expressed in the liver and the pancreas, and requires a high concentration of glucose to reach the maximum turnover rate. As a result of this, high glucose levels after food uptake are reduced by the production of glycogen in the liver [32]. The other hexokinase isozymes work with much lower substrate concentrations.

The main goal of the present work was to contribute to an evolutionary understanding of glycolysis by phylogenetic analyses of the 10 glycolytic enzymes from representatives of the vertebrate lineage. Based on the observation of increased size of gene families in vertebrates [10,33-40] and their highly specialized tissues, we expected to find duplications of entire pathways in the vertebrate lineage.

## Results

**Figure 2:**
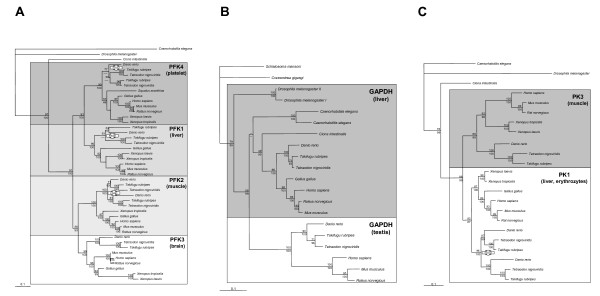
Maximum-likelihood tree of the tetrameric glycolytic enzymes phosphofructokinase (PFK), glyceraldehydes-3-phosphate dehydrogenase (GAPDH) and pyruvate kinase (PK) dataset comprising 44 amino-acid sequences for PFK (430 AA), 22 amino-acid sequences for GAPDH (340 AA), and 23 amino-acid sequences for PK (533 AA). Values at the branches are support values (ML bootstrapping/MB posterior probabilities). "FSGD" depicts putative fish-specific gene duplication events.

**Figure 3:**
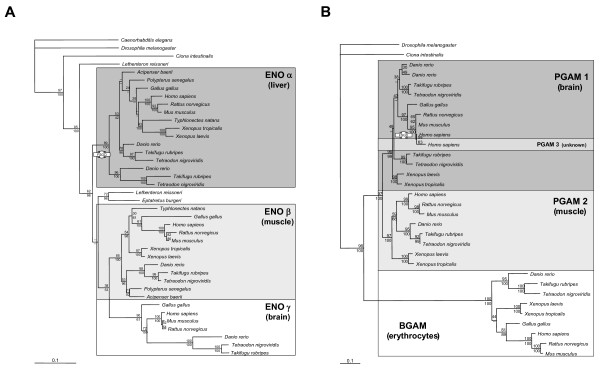
Maximum-likelihood tree of the heterodimeric composing glycolytic enzymes enolase (ENO), and phosphoglycerate mutase (PGAM) dataset comprising 40 amino-acid sequences for ENO (446 AA), and 32 amino-acid sequences for PGAM (256 AA). Values at the branches are support values (ML bootstrapping/MB posterior probabilities). 'FSGD' depicts putative fish-specific gene duplication events.

**Figure 4:**
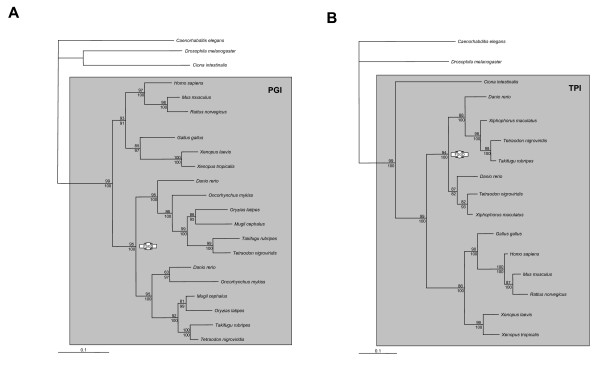
Maximum-likelihood tree of the homodimeric composing glycolytic enzymes phosphoglucose isomerase (PGI), and triosephosphate isomerase (TPI) dataset comprising 22 amino-acid sequences for PGI (555 AA), and 16 amino-acid sequences for TPI (250 AA). Values at the branches are support values (ML bootstrapping/MB posterior probabilities). 'FSGD' depicts putative fish-specific gene duplication events.

**Figure 5:**
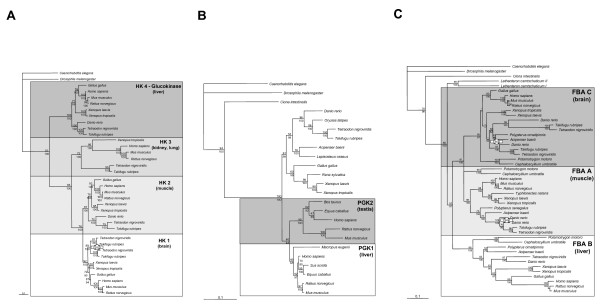
Maximum-likelihood trees of the monomeric glycolytic enzymes hexokinase (HK), phosphoglycerate kinase (PGK) and fructose-bisphosphate aldolase (FBA) dataset comprising 44 amino-acid sequences for HK (909 AA), 15 amino-acid sequences for PGK (417 AA), and 47 amino-acid sequences for FBA (366 AA). Values at the branches are support values (ML bootstrapping/MB posterior probabilities). 'FSGD' depicts putative fish-specific gene duplication events.

For most glycolytic enzymes, two or more copies can be found in vertebrates. The topologies for the inferred gene trees generally reflect the history of one or two rounds of duplications within the vertebrate lineage plus an additional duplication event within the teleost fish. The phylogenetic analyses confirm duplication events leading to multiple copies within vertebrates; these duplications occurred almost invariantly after the divergence of the urochordate *C. intestinalis *(Figures 2B, 2C, 3B, 4A, 4B, 5A, 5C)

### Tetrameric enzymes

Glycolytic enzymes, which are active as tetramers, occur as 1–4 copies in vertebrate genomes, likely as a result of ancient genome duplication events (1R and 2R). They display clearly different evolutionary patterns (Figure 2).

The tree for PFK reflects a perfect 1R/2R topology with three additional 3R events in the liver-specific isoform PFK1, the muscle-specific PFK2, and the platelet isoform PFK4 (Figure 2A). The first duplication led to PFK1/4 and PFK2/3 gene pairs (1R). The second duplication event segregates these precursors into the extant genes (2R). Except for PFK3, all PFK isoforms occur in more than one copy in ray-finned fishes (3R). However, for *Danio rerio*, searches of genomic and expressed sequence tag (EST) data yielded no second PFK1, PFK2 and PFK4 paralog as in the pufferfishes, where there is strong support for 3R. Since the *Danio rerio *genome is currently in a rather fragmented and incomplete state, the chances of missing data are quite high. On the other hand, the possibility of gene loss in certain lineages also cannot be neglected. Reciprocal loss of genes has been proposed as a mechanism for speciation [41].

The duplication of GAPDH seems to have occurred before the evolution of the bilaterian animals (Figure 2B). The liver-specific GAPDH (in vertebrates [42]) is found in all bilaterian species included in this analysis, whereas the testis-specific form occurs only in vertebrates. The tree topology of the liver-specific form reflects the general bilaterian phylogeny only in parts, most likely due to the sparse taxon sampling. Notably, the monophyly of protostomes and in particular the ecdysozoans is not recovered, since the two distinct copies of *Caenorhabditis *were placed as a sister group to the deuterostomes, albeit without significant support. For *Xenopus*, BLAST searches of genomic and EST data yielded no GAPDH copy.

The phylogeny of PK shows only one duplication event within the vertebrate lineage with an additional clearly resolved fish-specific duplication event, which occurred in the blood-specific [43] form PK1 (Figure 2C).

### Heterodimeric enzymes

The topologies for the obtained gene trees of ENO and PGM reflect the history of 1R/2R/3R (Figure 3). We obtained full-length ENO cDNA sequences for two genes each from bichir (*Polypterus senegalus*) and sturgeon (*Acipenser baerii*), both basal ray-finned fish, and caecilian (*Typhlonectes natans*). Database searches revealed three copies of ENO within the vertebrates (Figure 3A). The sequences of lampreys and hagfish cluster with the ENO γ paralogous group, implying that the first duplication (1R) took place before the split of cyclostomes from the gnathostome lineage, as it has also been indicated by a study on Hox genes [9]. The positions of another lamprey sequence is basal to the multiple copies, possibly a long-branch attraction artifact, pulling this fast-evolving sequence towards the outgroup. The liver-specific ENO α is duplicated in actinopterygians, with a proposed timing of the duplication before the divergence of *Polypterus *and *Acipenser*. The bootstrap support for this topology, which contradicts the current view of the fish-specific duplication being limited to teleosts, [44-46] is low. For Acipenserformes, however, polyploidy is a known phenomenon [47]. One fish-specific paralog displays an increased rate, especially in *Takifugu rubripes*. The differences in amino-acid sequence are distributed over the complete sequence and cannot be linked to a specific functional domain. The same is true for all three teleost ENO γ sequences used in this study.

The topology for PGAM reflects the well-supported history 2R/3R in the brain isoform PGAM1 and an additional gene duplication within the human lineage (Figure 3B). The first duplication led to erythrocyte-specific bisphophoglycerate mutase (BGAM) and the precursor of PGM1 and PGM2; the latter is assumed to be a muscle-specific isoform [48].

### Homodimeric enzymes

Within PGI and TPI, the major phylogenetic relationships are in agreement with the widely accepted phylogeny of vertebrates (Figure 4). Based on the phylogenetic analyses, duplication events leading to multiple copies within vertebrates could not be shown. However, there were duplication events during the evolution of ray-finned fish, so there are two copies each in zebrafish, puffer fishes, medaka, striped mullet and trout for PGI (Figure 4A), and two copies in zebrafish, platyfish and one pufferfish (*Tetraodon nigroviridis*) for TPI (Figure 4B), respectively. No second TPI paralog in *Takifugu rubripes *could be found within genomic and EST databases, which might indicate an event of gene loss.

### Enzymes only active as monomers

Figure 5 shows the ML trees of monomeric enzymes obtained in the phylogenetic analyses on the amino-acid level. Based on the phylogenetic analyses, duplication events leading to multiple copies during vertebrate evolution could be detected. The topology for HK shows three rounds of duplication within the vertebrate lineage, which is not in agreement with our expectations. An additional duplication event happened within the lineage of ray-finned fish in the brain isoform, HK1 (Figure 5A). The first duplication led to HK4 (glucokinase), a 50-kDa enzyme, and the protoortholog of HK1, 2, 3 (all 100 kDa). The second duplication produced HK3, which shows a somewhat higher rate of evolution than the other isoforms, and a HK1/2 precursor, which gave rise to HK1 and HK2 in a subsequent gene duplication that most likely occurred in a gnathostome ancestor (2R). Zebrafish paralogs for HK1 and HK 3 could not be found in the last version of the Ensembl database (WTSIZv5). Thus, the timing of duplication events within the ray-finned fish in HK1 cannot be determined, and the duplication might be limited to pufferfish species.

The analyses revealed a mammal specific duplication event for PGK (Figure 5B). They possess a testis-specific isoform (PGK2) and a liver-specific isoform (PGK1). The position of the wallaby sequence implies that the duplication occurred before the divergence of placental mammals and marsupials.

Based on the phylogenetic analyses, the FBA duplication events leading to the multiple copies within vertebrates occurred clearly after the divergence of the lampreys (Figure 5C), which suggests a timing of the 1R/2R after the cyclostome split (but see the ENO tree, Figure 3B). The brain-specific isoform FBA C and the muscle-specific isoform FBA A show additional duplication events within the ray-finned fish lineage. For FBA C within the teleosts, a duplication preceding the split of *Polypterus *and *Acipenser *is proposed; this is not in agreement with the current hypothesis of the timing of the FSGD [44-46]. The unexpected topology is probably caused by a reconstruction artifact due to the very fast-evolving sequences of one of the fish-specific copies. A study based on yeast paralogs has shown that an increased evolutionary rate of one copy can lead to errors in phylogenetic reconstruction [49]. The differences in the sequences are distributed over the complete coding sequences and not restricted to a specific domain. The remaining sequences do resemble the general expectations of vertebrate phylogenetic relationships [50]. We also obtained FBA sequences for *Acipenser baerii *and *Polypterus senegalus *that clustered in the paralog A group, which is considered to be the muscle-specific isoform. One additional copy of FBA A in *Danio rerio *placed basal to the zebrafish/pufferfish split rejects the possibility of a zebrafish-specific duplication event. The *Typhlonectes natans *(caecilian) sequence (FBA A) forms a monophyletic group with the sequences from the *Xenopus *species, as expected. The FBA B isoform places the basal ray-finned fish (*Acipenser baerii, Polypterus ornatipinnis*) basal to a cluster containing tetrapods and derived ray-finned fish (*Danio rerio*, *Tetraodon nigroviridis*). This might be due to the partial character of these sequences, which were used from a previous study [29].

## Discussion

The individual glycolytic enzymes are among the most slowly evolving genes [51], yet the glycolytic pathway has adapted to the varying metabolic requirements of different tissues and different organisms. Genome duplications appear to have been the principal mechanism that gives rise to multiple copies of isoenzymes. The topologies for eight of the gene trees (Figures 2, 3, 4, 5) generally reflect the 1R/2R/3R genome duplication history during vertebrate evolution. Convincing data supporting the 2R hypothesis stems from paralogons, genomic regions containing paralogous genes and therefore being the result of large-scale duplications [52-54]. Only some of the glycolytic enzymes showing 1R/2R duplications are found on chromosomes where paralogons have been previously reported, i.e., PK (PK3 on chromosome 15, PK1 on chromosome 1), ENO (ENOα on chromosome 1, ENOβ on chromosome 17, ENOγ on chromosome 12), HK (HK1 on chromosome 10, HK2 on chromosome 2, HK3 on chromosome5), and FBA (FBAA on chromosome 16, FBAC on chromosome 17).

For many single-copy genes in tetrapods, two copies have been described for ray-finned fish. The first observation of this pattern began with the discovery of more than four Hox clusters in zebrafish (*Danio rerio*) [55] and medaka (*Oryzias latipes*) [56]. Recent data from puffer-fish genomes confirmed the existence of at least seven Hox clusters even in these very compact genomes [57,58]. With an increase of available sequences, especially from genome and EST projects, the number of genes which show a duplication event in the fish lineage increased significantly [10-12,15,34,38,59-61]. Data from the genes analyzed in this study, including genomic sequences (*Tetraodon nigroviridis*, *Takifugu rubripes*) and EST data (*Danio rerio*), shows that enzyme isoforms were duplicated before the divergence of Ostariophysii (zebrafish) and Neoteleostei (medaka, pufferfishes). The determination of the phylogenetic timing of the duplication event for glycolytic genes is difficult due to missing sequence data for basal actinopterygian species (bichir, sturgeon, gar and bowfin). Also, in many cases a strikingly increased evolutionary rate of at least one copy of the duplicated genes might result in a basal position of this paralogous cluster via LBA artifacts ("outgroup tree topology"). [49,62] rendering the phylogenetic reconstruction of the ancient events (~400-350 MYA) difficult [63]. Previous studies have shown that the most likely position of the 3R genome duplication event is after the divergence of gar/bowfin (Holostei) from the teleost lineage [44-46].

### Hexokinase

Glycolytic enzymes are often expressed in a tissue-specific manner. For example, the different types of vertebrate HK (Figure 5A), each with distinct kinetic properties, are expressed in different kinds of tissue. HK 1 is the predominant isoenzyme in the vertebrate brain, HK 2 predominates in muscle tissue, and HK 4 in hepatocytes and pancreatic islets. The kinetic properties of these three isoenzymes are well adapted to the roles of glucose phosphorylation in the different cell types [64]. Both HK 1 and HK 2 are saturated at glucose concentrations in the normal physiological range for blood, and thus their kinetic activity is largely unaffected by variations. When the availability of glucose is pathologically low, it is more important to satisfy the glucose needs of the brain than those of other tissues, and a low K_m _of HK 1 allows it to perform at low glucose concentrations. The kinetic behavior of HK 4, which requires high concentrations of glucose for maximal activity, is very different, but this is in agreement with functions in liver and pancreas cells as regulators of blood-glucose concentration [65,66]. The function of HK 3 is inhibited by excess glucose [67], the reason for this is still not fully understood.

Based on the phylogeny reconstructed here (Figure 5A) as well as previous reports [64], HK 4 is the oldest member of this gene family. HK 4 consists of a 50-kDa fragment, whereas the other HKs have a size of 100 kDa. A more detailed analysis with separately considered amino and carboxy termini suggests that a fusion event led to the present isoenzymes [64]. We were also able to document a fish-specific duplication of HK 1, however, nothing is known about possible functional consequences due to their duplication in terms of sub- or neofunctionalization.

### Phosphoglucose isomerase

PGI is a multifunctional protein, also known as neuroleukin (NLK), autocrine mobility factor (AMF), or differentiation and maturation mediator. Although it was proposed that the multiple functions of PGI were gained gradually by amino-acid changes [68], an alternative hypothesis is that PGI is recruited by other proteins for novel functions during evolution [69]. Two lines of evidence support this hypothesis. First, the protein is highly constrained, and second, *Bacillus *PGI not only can replace the glycolytic aspects of the enzyme, but also fulfil NLK and AMF functions in mammalian cells[70,71]. The multiple functions were proposed to be innate characteristics of PGI at the origin of the protein [69]. The novel functions of PGI might have evolved by cellular compartmentalization of the protein, dimerization, and evolution of its receptors. The enzyme is found to be active as a dimer in glycolysis. It is not clear whether it is active in its other functions as a monomer or as an oligomer. This multi-functionality and the possible function as an oligomer might explain the retention of two copies in the fish lineage. The topology (Figure 4A) suggests that the only gene-duplication event of PGI occurred in ray-finned fish before the diversification of Acanthopterygii but after the split of ray-finned fish and tetrapods.

### Phosphofructokinase

The PFK gene family is composed of four different genes (Figure 2A): They are expressed in liver (PFK1), muscle (PFK2), brain (PFK3) and platelets (PFK4) [27]. These genes differ both in size and physico-chemical properties, and are also expressed in varying amounts in different tissues. PFK occurs in a variety of oligomeric forms from dimer to tetramer to octamer and even larger forms. The vertebrate enzyme, however, is active as a tetramer. Because the subtypes can associate randomly, each tissue contains not only homotetrameric enzymes, but also various types of heterotetramers. These different assemblies of subunits result in complex isoenzymic populations with a wide variety of kinetic properties [72]. It seems likely that the copies result from 2R. The number of possibilities of PFK combinations in ray-finned fish is even higher because of 3R (PFK1, PFK2, PFK4). The functional significance of the complicated quaternary structure of PFK is not entirely clear, but probably relates to the requirement for specific responsive control properties for this enzyme. A wide range of effector molecules have been described [73-75], and some forms of the enzyme can be also regulated by phosphorylation [76-78].

### Fructose-bisphosphate aldolase

The three FBA isoenzymes A, B, C in vertebrates [79] also have a tissue-specific distribution [80,81]. FBA A, which is the most efficient in glycolysis, is the major form present in muscle. FBA B seems to function in gluconeogenesis and is only expressed in liver and kidney, where it is the predominant form. FBA C, with intermediate catalytic properties, is found in the brain. In the FBA tree (Figure 5C), the lamprey sequences preceded the first duplication, while the Agnatha clade in the ENO analyses (Figure 3A) clusters with one branch of the duplication. Statistical support for the nodes around 2R and the divergence of cyclostomes, however, is high. Multiple sequences from Chondrostei (sharks and rays) for FBA, which are clearly grouped with the three paralogous groups, suggest a timing of the duplications before their separation from the Osteichthyes lineage. Within the fish lineage, FBA A was duplicated before the divergence of Ostariophysii (zebrafish) and Neoteleostei (medaka, pufferfish). However, in the FBA C subtree, gar and bichir are grouped within one paralogous group. Either one paralogous copy for gar and bichir of this gene has not been found yet, or this reconstruction is due to a reconstruction artifact caused by the extremely fast-evolving sequences of the teleost sequences (zebrafish and pufferfishes), which get drawn to the basis (LBA).

### Triosephosphate isomerase

TPI is highly conserved in sequence, structure, and enzymatic properties [82]. The enzyme is functional as a homodimer. The topology (Figure 4B) suggests that the only gene-duplication event of TPI occurred in ray-finned fish before the diversification of Acanthopterygii but after the split of ray-finned fish and tetrapods. This corroborates the results of a previous study [83] supporting a single gene duplication event early in the evolution of ray-finned fish. Comparisons between inferred ancestral TPI sequences indicated that the neural TPI isozyme evolved through a period of positive selection, resulting in the biased accumulation of negatively charged amino acids. If both copies are coexpressed, TPI could act as heterodimer in fish with consequences in specificity or enzyme kinetics.

### Glyceraldehyde-3-phosphate dehydrogenase

GAPDH is the most highly conserved of all glycolytic enzymes. The rate of evolution of the catalytic domain, for example, is only 3% per 100 million years [27]. Thus, these domains in eukaryotic and eubacterial enzymes are >60% identical. Due to this constraint we had to include basal animal lineages (arthropods, flatworms, nematodes and mollusks) into the analysis to clearly identify the origins of two copies of GAPDH (Figure 2B). The GAPDH acts as a tetramer, however, it is not clear whether this is constituted out of two different isoenzymes in vertebrates similar to the PFK composition. There is evidence for an ancient duplication around the bilaterian origin; however, the testis-specific copy was found only in vertebrates, which makes this scenario rather unlikely. It has been hypothesized that vertebrates acquired a second copy, only expressed in the testis, by retroposition [84,85]. However, many more new gene copies were created, most of which, if not all, seem to be pseudogenes [42,86,87]. This might be also the case for the muscle-specific form, which only occurs in primates. Despite the possibility of requiring variability by composing heterotetramers with additional isoenzymes, it is also possible that paralogs are retained because GAPDH is also involved in the maintenance of specific subcellular structures, e.g. the bundling of microtubules [88].

### Phosphoglycerate kinase

The quaternary structure of most glycolytic enzymes has been well conserved during evolution. Monomeric forms are unusual, and one enzyme that is invariably a monomer is phosphoglycerate kinase. In mammals, two different, but functionally similar isoenzymes for phosphoglycerate kinase have been detected. One form occurs in all somatic cells predominantly in the liver. The other form is only found in sperm cells [89]. The gene for the major isoenzyme (*pgk1*) is X-linked. Expression of this gene coincides with overall activity of the X chromosome. Its transcription is thus constitutive, regardless of the cell type, when the chromosome is active. When spermatogenic cells enter meiosis, the X chromosome is inactivated and the second gene (*pgk2*), which is autosomal (chromosome 6 in humans), is expressed [90]. It has been proposed that the *pgk2 *gene, which does not contain any introns in contrast to *pgk1*, must have evolved from the *pgk1 *gene by retroposition [89,91]. Our phylogenetic analysis suggests that this must have happened early in mammalian evolution (Figure 5B). Although weakly supported, the position of the wallaby sequence (*Macropus eugenii*) implies that the duplication occurred before the divergence of placental mammals and marsupials.

### Phosphoglycerate mutase

In the cofactor-dependent PGAM gene family, three paralogs can be found in all vertebrates. These isoenzymes are expressed in a tissue-specific manner and have been classified as brain (PGAM1), muscle (PGAM2) and erythrocyte (BGAM) types. In some tissues, more than one gene is active, resulting in multiple isoenzymes composed of homo- and heterodimers [92]. The phylogenetic analyses (Figure 3B) shows that the three isoenzymes found in vertebrates have evolved from a common ancestor by two separate gene-duplication events. A PGAM3 form was proposed in human and chimp [93], probably as a result of primate-specific gene duplication. Our findings suggest that a more recent duplication gave rise to the PGAM1 and PGAM2 copies. BLAST searches against the chicken genome detected only the PGAM1 form. This could be explained by gene loss of the PGAM2 gene in the avian line, or by the incompleteness of the genome assembly. In our phylogeny, the origin of PGAM predates the PGAM1 and PGAM2 divergence. This clarifies uncertainties of previous studies in unravelling the evolutionary history of PGAM [27,48]. Vertebrate PGMs are rather versatile and can catalyze three different reactions (they act as mutase, synthase or phosphatase). Initially it was supposed that each of these reactions was catalyzed by a different enzyme, and it was quite surprising when it was realized that the PGM could each catalyze all three of these reactions, albeit at substantially different rates [94]. One can speculate that these differences in activity rates acted in favor of the maintenance of several copies during evolution.

### Enolase

For ENO three different isoenzymes also occur in vertebrate tissues, termed α, β and γ. The active enzyme is a homo- or heterodimer. The α form is present in many tissues, especially in the liver, β predominates in muscle and γ is only found in brain cells. The topologies for the gene tree generally reflect the history of 2R/3R for ENO α (Figure 3A). However, the position of the Cyclostomata sequences is not consistent and therefore offers no information about the relative timing of the duplication events. One lamprey sequence precedes the first duplication, while the Agnatha clade in the ENO β analyses clusters with one branch of the duplication, however, there is very little support. This is not in agreement with the current hypothesis of the relative timing of 2R [9]. Two functions have been attributed to ENO in addition to its normal catalytic activity. First, ENO plays a structural role in the eye lens. A major lens protein of lampreys, some fishes and birds is τ-cristallin. This protein and α-ENO appear to be identical [95-97]. The additional duplication within the fish lineage in ENO α might provide a bigger "toolbox" for this gene's function while retaining its glycolytic pathway role simultaneously. The additional role that ENO may fulfill is the acquisition of thermal tolerance [98]. The Enolase genes are positioned in well described paralogons of the human genome on chromosomes 1 (ENO α), 17 (ENO β) and 12 (ENO γ) [53], This implies that they are resulting from a large-scale duplication event, probably a genome duplication.

### Pyruvate kinase

It was originally expected that PK had four different isoforms encoded by four different genes. However, it is known now that there are only two different genes: one encoding the PK3 (m-form) isoforms and one for the PK1 (l and r forms) isoenzymes. Additional isoenzymes can arise from differential RNA splicing. Therefore, the phylogeny (Figure 2C) is only considering one gene product for each isoenzyme. The differences between the spliced isoforms are too small to include into a phylogenetic analysis. Both copies seem to be derived from a duplication event in early vertebrate history (1R or 2R) and are expressed in a tissue-specific manner. PK1 is the most abundant form in liver, where gluconeogenesis plays an important role [99]. PK3 is the major form in tissues, where glycolysis predominates such as muscle, heart and brain. Both isoenzymes show different enzyme kinetics according to their occurrence. The PK is active as a tetramer, which is regulated by the thyroid hormone and fructose 1,6-bisphosphate [100,101]. Usually PK is active as homotetramer but in some cases, it also acts as a heterotetramer. This might be an explanation for why the copies of the fish-specific duplication in PK1 were retained during evolution. As shown previously, the increase in possible combinations of heterotetramers leads to increased specificity in enzyme kinetics.

## Conclusion

From our data, we could not detect a 1R/2R/3R trend consistent for all enzymes of the glycolytic pathway. Even though most of them do show a repeated pattern of duplications, which are accompanied by tissue-specific expression, this is not the case for all of them. Considerations of tertiary protein structure also could not give further indications for why some enzymes have four isozymes in tetrapods and others only one. Given the expectation that most genes get lost rather rapidly after a duplication event [17,18], the tissue-specific expression might have led to an increased retention for some genes since paralogs can subdivide the ancestral expression domain (subfunctionalization) or find new functions, which are not necessarily related to the original function (neofunctionalization [95]). This is, however, not true for all genes, and we can conclude that the pathway is not evolving as a unit but each gene follows its own history, as has been shown previously for Bacteria and Archaea [25,26]. For a better understanding of the gene-duplication history, further genome projects on a greater diversity of evolutionary lineages will be required.

## Methods

### Sequencing

ENO and FBA cDNAs for bichir *Polypterus ornatipinnis*, sturgeon *Acipenser baerii *and caecilian *Typhlonectes natans *were sequenced using degenerated primers designed based on amino-acid alignments of previously known sequences and the rapid amplification of cDNA end (RACE) method to obtain complete coding sequences. Total RNA was extracted from muscle tissue freshly frozen in liquid nitrogen and stored at -80°C. Extractions were performed with Trizol (Gibco, Germany). cDNA first strand syntheses were done using the First Strand synthesis kit following the manufacturers manual (Gibco, Germany). A c-tailing step was added to allow 5' RACE. Fragments were amplified using degenerate primers based on the amino-acid sequences of previously reported sequences. See Table 1 for sequences of degenerate primers. Amplification was performed in 50-μl reactions containing 0.5 units of RedTaq (Sigma, Germany), RedTaq reaction buffer (10 mM Tris-HCl, pH 8.3, 50 mM KCl, 1.1 mM MgCl_2_, 0.01% gelatin), 0.2 μM of each primer (MWG-Biotech AG, Germany, 0.4 mM dNTPs (Peqlab Biotechnology, Germany) and 0.5 mM MgCl_2_. Cycle conditions included an initial denaturation step of 94°C, then 35 cycles of 94°C for 10 seconds, 42°C for 1 minute and 72°C for 2 minutes. Final extension was performed at 72°C for 5 minutes. PCR products were purified either directly or, in cases of multiple bands, by cutting bands from 1% agarose gels and using the QIAGEN spin system. 3' RACE reactions were performed with nested approaches of two sequence-specific primers and the Not-I short primer (AAC TGG AAG AAT TCG CGG CC). 5'RACE were preformed with nested sequence specific primers and the oligo-G primer binding the c-tail at the 5' end of the cDNA (CTA GTA CGG GII GGG IIG GG). Sequences were confirmed by amplification and sequencing of both strands of the complete coding sequences by specific primers located in the 5' and 3' non-coding regions. Cycle sequencing was performed using the ABI sequencing mix and 35 cycles of 94°C for 10 seconds, 42°C – 50°C for 10 seconds and 68°C for 4 minutes. Sequences were run on an ABI3100 capillary sequencer. Sequences were proofread and assembled using Sequence Navigator [102].

### Database searches and sequence analyses

Protein sequences of pufferfishes (*Tetraodon nigroviridis*, *Takifugu rubripes*) zebrafish (*Danio rerio*), human (*Homo sapiens*), mouse (*Mus musculus*), rat (*Rattus norvegicus*) chicken (*Gallus gallus*), claw frogs (*Xenopus laevis, Xenopus tropicalis*), sturgeon (*Acipenser baerii*), caecilian (*Typhlonectes natans*), bichir (*Polypterus sp*.), lamprey (*Lethenteron sp, Eptatretus burgeri*), shark (*Cephaloscyllium umbratile*), and ray (*Potamotrygon motoro*) were obtained from the Ensembl database [103] or by conducting BLAST (BLASTp and translated BLAST) searches [104] against GenBank. All accession numbers are listed in the supplementary data. Sequences were aligned with Clustal X [105]. For each alignment, a preliminary tree was drawn. This tree facilitated the identification of identical sequences, sequences that varied only in length, and multiple sequences within species that differed by only few amino acids, all of which were removed from the alignment. Draft trees were reconstructed from the remaining sequences using Poisson-corrected genetic distances and the neighbor-joining algorithm [106] in MEGA 3.0 [107]. If subsequent phylogenetic surveys provided an indication for fish-specific gene duplication, additional BLAST searches were conducted to find more putative actinopterygian copies. With a few exceptions, human "reference sequences" [108] were used as query sequences for the BLAST searches. Species were surveyed one at a time to improve the identification of a drop in sequence similarity, which was used as a "cut-off" criterion.

As outgroup sequences, we used data from *Caenorhabditis elegans*, *Drosophila melanogaster *and *Ciona intestinalis*. In one case (GAPDH), we used data from *Schistosoma mansoni *and *Crassostrea gigas *as outgroup sequences. In another case (PGK), we extended the dataset with protein sequences from *Oryzias latipes*, *Lepisosteus osseus*, *Rana sylvatica*, *Equus caballus*, *Sus scrofa*, *Bos taurus *and *Macropus eugenii*. Amino-acid data were analyzed using PHYML [109] and the maximum-likelihood (ML) model, and parameters were chosen based on ProtTest [110] analyses. Confidence in estimated relationships of ML tree topologies was evaluated by a bootstrap analysis with 500 replicates [111] and Bayesian methods of phylogeny inference. Bayesian analyses were initiated with random seed trees and were run for 200,000 generations. The Markov chains were sampled at intervals of 100 generations with a burn-in of 1000 trees. Bayesian phylogenetic analyses were conducted with MrBayes 3.1.1 [112].

## Authors' contributions

DS designed the study, carried out the phylogenetic analyses, and drafted the manuscript. SH conceived the study, carried out the molecular work, participated in the phylogenetic analyses and drafted the manuscript. HB participated in the phylogenetic analyses, and helped to draft the manuscript. AM participated in the study design and coordination and helped to draft the manuscript. All authors read and approved the final manuscript.

## Supplementary Material

Additional File 1 A complete list of GenBank, JGI, and Ensembl accession numbers of the amino acid sequences used for the phylogenetic analyses of this study is provided in the file Click here for file

## Figures and Tables

**Table 1:** Degenerate primers designed and used in this study

Name	Sequence	Enzyme
Eno.uni.FN1	GGN AAY CCI ACI GTN GAR GT	Enolase
Eno.uni.FN2	ACI GGI ATH TAY GAR GC	Enolase
Eno.uni.FM	AAR TAY GGI AAR GAY GC	Enolase
Eno.uni.RC1	GT RTC YTC IGT YTC NCC	Enolase
Eno.uni.RC2	GC ICC IGT YTT DAT YTG	Enolase
Eno.uni210.F	ACC AAC GTT GGI GAY GAR GGI GG	Enolase
Eno.uni320.R	TTG GTC ACA GTI ARR TCT TCI CC	Enolase
FBA.Super+	GGT AAR GGT ATC YTI GCI GCI GAY G	Fructose-Bisphosphate Aldolase
FBA.uni.125+	ACT ACT AYT CAG GGN YTN GAY GG	Fructose-Bisphosphate Aldolase
FBA.uni.240-	GC ATG ACC AGS AGT NAC CAT RTT NGG	Fructose-Bisphosphate Aldolase
FBA.uni.315-	CAG AGC AGW AGC CTG CAG NGC NCK NCC	Fructose-Bisphosphate Aldolase
