# Supplementary material for: Three rounds (1R/2R/3R) of genome duplications and the evolution of the glycolytic pathway in vertebrates
Source: BMC Biol. 2006 Jun 6;4:16. doi: 10.1186/1741-7007-4-16 (PMC1508162; doi:10.1186/1741-7007-4-16)
Supplement: Additional File 1 — A complete list of GenBank, JGI, and Ensembl accession numbers of the amino acid sequences used for the phylogenetic analyses of this study is provided in the file [file 1741-7007-4-16-S1.pdf]

Supplementary material: GenBank, JGI, ENSEMBL accession numbers of the amino acid sequences used for the phylogenetic analyses.

Hexokinase (HK)	
Homo sapiens	P19367, P52789, P52790, P35557
Mus musculus	P17710, O08528, XP_127381, P52792
Gallus gallus	NP_989432, NP_989543, AAM83106
Rattus norvegicus	P05708, P27881, P27926, P17712
Xenopus laevis	AAH72832, BQ734748, 2210326
Xenopus tropicalis	ENSXETP00000018649, ENSXETP00000019458, ENSXETP00000041217, ENSXETP00000042536
Danio rerio	ENSDARP00000049485, ENSDARP00000024665
Takifugu rubripes	SINFRUP00000130243, SINFRUP00000175342, SINFRUP00000171538, SINFRUP00000132593, SINFRUP00000154946
Tetraodon nigroviridis	GSTENP00031116001, GSTENP00029591001, GSTENP00025793001, GSTENP00004899001, GSTENP00025792001
C. elegans	F14B4.2a.1
D. melanogaster	CG3001

Phosphoglucose isomerase (PGI)	
Homo sapiens	AAH04982
Mus musculus	AAH88995
Gallus gallus	NP_00100612
Rattus norvegicus	AAH62005
Xenopus laevis	AAH73315
Xenopus tropicalis	AAH90090
Danio rerio	CAC83782, AAH44450
Mugil cephalus	CAC83778, CAC83779
Oncorhynchus mykiss	BX083050, BX075627
Oryzias latipes	sca751, sca4041
Takifugu rubripes	SINFRUP00000145974, SINFRUP00000159975
Tetraodon nigroviridis	CAG01218, CAG14252
C. elegans	NP_493380.1
D .melanogaster	CG8251

Phosphofructokinase (PFK)	
Homo sapiens	P16118, O60825, Q16877, Q01813
Mus musculus	AAH57594, Q7TS91, P70265, XP_284750
Gallus gallus	Q91348, XP_414257, XP_417979, XP_416472
Rattus norvegicus	P47858, Q9JJH5, P47860, P30835
Xenopus laevis	AAH70579, AAH84893, AAH70776,
Xenopus tropicalis	ENSXETP00000037288, ENSXETP00000039305, ENSXETP00000020639, ENSXETP00000047683
Danio rerio	ENSDARP00000020245, ENSDARP00000036459, ENSDARP00000065761, ENSDARP00000020946
Takifugu rubripes	SINFRUP00000143959, SINFRUP00000154096, SINFRUP00000159206, SINFRUP00000162973, SINFRUP00000157362, SINFRUP00000166112, SINFRUP00000145680
Tetraodon nigroviridis	GSTENP00006196001, GSTENP00013641001, GSTENP00027279001, GSTENP00023398001, GSTENP00013249001 GSTENP00023654001 GSTENP00013249001
C. elegans	Q27483
D.melanogaster	P52034
Ciona instestinalis	ENSCINP00000010042

Supplementary material: GenBank, JGI, ENSEMBL accession numbers of the amino acid sequences used for the phylogenetic analyses.

**Fructose-bisphosphate aldolase (FBA)**	
Homo sapiens	AF054987, M11560, X02747
Mus musculus	BC008184, Y00516, BC036132
Gallus gallus	BX929868, M10946
Rattus norvegicus	M63656, M14420, M10149
Xenopus laevis	AAB31152, BAA19524, AAH44676
Xenopus tropicalis	AAH67946, AAH74643, AAH61442
Acipenser baerii	AB111405, AB111404, AB111403, DQ497981
Cephaloscyllium umbrat.	BAD17931, BAD17932, BAD17933
Danio rerio	BC053192, AY394966, AY394965, XP_707430, XP_690284
Lethen. camtschaticum	D38620, D38619
Polypterus ornatipinnis	BAD17924, BAD17925, BAD17926, DQ497983
Potamotrygon motoro	BAD17938, BAD17939, BAD17940
Takifugu rubripes	SINFRUP00000154393, SINFRUP00000144527, SINFRUP00000136751
Tetraodon nigroviridis	CAG0049, CAG0759, CAG0627, CAG0895
Typhlonectes natans	DQ497982
C. elegans	NP_741281
D. melanogaster	AAS65220
Ciona intestinalis	Scf235

**Triosephosphate isomerase (TPI)**	
Homo sapiens	NM_000365
Mus musculus	NM_009415
Gallus gallus	NM_205451
Rattus norvegicus	NM_022922
Xenopus laevis	BC046864
Xenopus tropicalis	ENSXETP00000043527
Danio rerio	AF387820, AF387819
Oryzias latipes	AB183488, AB111387
Takifugu rubripes	AL834781
Tetraodon nigroviridis	CAA1007830, GSTENP00005289001
Xiphophorus maculatus	AAK85205, AAK85204
C. elegans	NM_064162
D .melanogaster	NM_176589
Ciona intestinalis	ENSCINP00000013486

**Glyceraldehyde-3-phosphate dehydrogenase (GAPDH)**	
Homo sapiens	P04406, O14556 P00354
Mus musculus	NP_032111, P16858
Gallus gallus	NP_989636
Rattus norvegicus	NP_076454, NP_058704
Danio rerio	ENSDARP00000049709, ENSDARP00000009462
Takifugu rubripes	SINFRUP00000156120, SINFRUP00000142721
Tetraodon nigroviridis	GSTENP00035345001, GSTENP00015338001
Crassostrea gigasgi	CAD67717
Schistosoma mansoni	P20287
C. elegans	K10B3.7.1, T09F3.3.1
D. melanogaster	CG8893, P07486
Ciona intestinalis	ENSCINP00000005672

Supplementary material: GenBank, JGI, ENSEMBL accession numbers of the amino acid sequences used for the phylogenetic analyses.

**Phosphoglycerate kinase (PGK)**	
Homo sapiens	Q8NI87, NP_620061
Mus musculus	P09411, AAH61054
Gallus gallus	P51903
Rattus norvegicus	AAH6316, XP_576525
Equus caballus	P00559 Q8MIF
Sus scrofa	Q7SIB7
Bos taurus	XP_581328
Macropus eugenii	P29408
Xenopus laevis	AAH43781
Xenopus tropicalis	CR760747
Rana sylvatica	Q9DG72
Danio rerio	AAH65888
Oryzias latipes	BAD17900
Takifugu rubripes	SINFRUP00000160355
Tetraodon nigroviridis	GSTENP00014631001
Lepisosteus osseus	BAD17907
Acipenser baerii	BAD17922
Ciona intestinalis	ENSCINP00000004571
C. elegans	NP_491245
D. melanogaster	CG3127

**Phosphoglycerate mutase (PGAM)**	
Homo sapiens	NP_002620, NP_000281, AAA51840
Mus musculus	NP_075907, NP_061358 BAC31541
Gallus gallus	CAG31362, CAG32689
Rattus norvegicus	NP_445742,NP_059024 AAH62240
Xenopus laevis	AAH70630, AAH82455 AAH73185
Xenopus tropicalis	NP_001004858, AAH74692, AAH67978
Danio rerio	ENSDART00000016350, ENSDART00000008287, NP_957318, NP_001002630
Takifugu rubripes	SINFRUP00000143399, SINFRUP00000144165, SINFRUP00000168929, SINFRUP00000139469
Tetraodon nigroviridis	GSTENP00029715001, GSTENP00016506001, GSTENP00026289001, GSTENP00011651001
C. elegans	NP_492409
D. melanogaster	NP_524546
Ciona intestinalis	ENSCINP00000016603

**Enolase (ENO)**	
Homo sapiens	P06733, P09104, P13929
Mus musculus	P21550, P17182, P17183
Gallus gallus	P51913, P07322, P51913
Rattus norvegicus	P04764, P07323, P15429
Xenopus laevis	AAH45082, CAA68706
Xenopus tropicalis	AAH96516, AAH61287
Acipenser baerii	DQ497984,DQ497987
Danio rerio	AAH59434, AAM88901, AAH72713, AAQ97775
Eptatretus burgeri	BAA88479
Lethenteron reissneri	BAA88483, BAA88482
Polypterus senegalus	DQ497985, DQ497988
Takifugu rubripes	SINFRUP00000150208, SINFRUP00000131536, SINFRUP00000156170, SINFRUP00000169571
Tetraodon nigroviridis	CAF8980, CAF9382, CAG0691, CAF9063
Typhlonectes natans	DQ497986, DQ497989
C. elegans	NP_001022349
D. melanogaster	EAL33991
Ciona intestinalis	ENSCINP00000015064

Supplementary material: GenBank, JGI, ENSEMBL accession numbers of the amino acid sequences used for the phylogenetic analyses.

**Pyruvate kinase (PK)**	
Homo sapiens	NP_000289, NP_872270
Mus musculus	ENSMUSP00000034834,<br>ENSMUSP00000035417
Gallus gallus	P00548
Rattus norvegicus	ENSRNOP00000015398,<br>ENSRNOP00000027700
Xenopus laevis	Q92122, AAH60485
Xenopus tropicalis	ENSXETP00000011367,<br>ENSXETP00000028820
Danio rerio	ENSDARP00000006300,<br>ENSDARP00000017190,<br>ENSDARP00000061565
Takifugu rubripes	SINFRUP00000128238,<br>SINFRUP00000129186,<br>SINFRUP00000141035
Tetraodon nigroviridis	GSTENP00012066001,<br>GSTENP00015375001,<br>GSTENP00025632001
C. elegans	NP_492458
D. melanogaster	O62619
Ciona intestinalis	ENSCINP00000008552
